# Functionalized Metal–Organic Framework Thin Films for Stable and Efficient Electrochemical Water Oxidation under Near-Neutral Conditions

**DOI:** 10.1021/acselectrochem.6c00031

**Published:** 2026-04-15

**Authors:** Sumanta Basak, Arshia Sulaiman, Amanda J. Morris

**Affiliations:** † Department of Chemistry, Virginia Polytechnic Institute and State University, Blacksburg, Virginia 24061, United States; ‡ Macromolecules Innovation Institute, 1757 Virginia Polytechnic Institute and State University, Blacksburg, Virginia 24061, United States

**Keywords:** water oxidation, ruthenium catalyst, metal−organic frameworks, electrocatalysis, thin-film electrodes

## Abstract

The development of molecularly modified metal–organic frameworks (MOFs) for electrochemical water oxidation has emerged as a promising strategy for efficient artificial photosynthesis. In this study, a ruthenium-based water oxidation catalyst (WOC) was incorporated into the UiO-67 framework, forming RuM–UiO-67 films grown directly on conductive FTO substrates. These modified films demonstrate efficient water oxidation activity at near-neutral pH (pH 6), operating at a low overpotential of ∼600 mV. The catalyst exhibits a turnover frequency (TOF) of (0.32 ± 0.02) s^–1^ at 1.5 V versus the normal hydrogen electrode (NHE) in buffered solution (pH 6) for the oxygen evolution reaction. Notably, incorporation into the MOF results in a 12-fold increase in electroactive surface coverage compared to a monolayer of the same catalyst on bare FTO. Faradaic efficiency analysis revealed incomplete conversion to O_2_, and follow-up iodometric analysis confirmed the formation of H_2_O_2_ as a competing two-electron oxidation product during electrocatalysis. These results highlight the utility of MOF-based architectures for maximizing the catalyst accessibility and stability under electrochemical water oxidation conditions.

## Graphical abstract

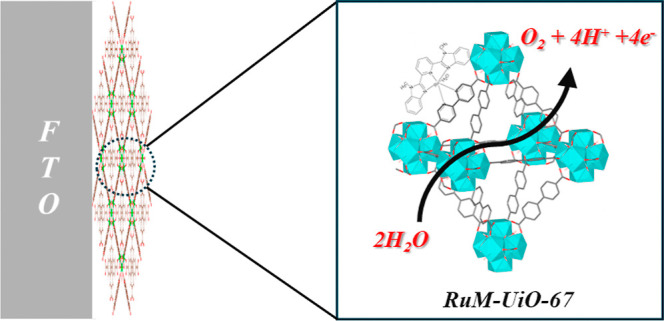

## Introduction

The adaptation of sustainable energy sources is one of the most pressing scientific and technological challenges of the 21st century. Among the various strategies for renewable energy conversion and storage, artificial photosynthesis, which directly transforms solar energy into chemical fuels through water splitting, holds great potential for providing a carbon-neutral solution to future energy demands.^1^ A crucial step in the process is the oxygen evolution reaction (OER) that is kinetically sluggish and thermodynamically not favorable because it needs the simultaneous removal of four electrons and four protons as well as the formation of the O–O bond.^2^ The development of efficient, stable, and earth-abundant water oxidation catalysts (WOCs) remains a key bottleneck in enabling large-scale solar fuel production. Many transition metals, including iridium, iron, and cobalt, are known to function as WOCs. Ruthenium, in particular, has garnered significant attention over the past 40 years for several reasons. First, ruthenium’s prominence stems from the availability of synthetically accessible complexes. Its relatively slow ligand exchange rates also increase the likelihood of observing intermediates. Additionally, ruthenium can exist in various oxidation states (from +1 to +8 and −2).^3,4^ Versatility in oxidation states enables ruthenium complexes to effectively cycle through different states, facilitating the multistep water oxidation process. Moreover, ruthenium complexes are stable and active in acidic environments.^5^ Early work, such as that with the well-known “blue dimer” reported by Meyer and co-workers, established the viability of Ru-based WOCs and has since spurred extensive studies into mononuclear complexes. These molecular systems often demonstrate low overpotentials and high turnover frequencies in homogeneous conditions.^6^

While homogeneous water oxidation catalysts (WOCs) play a significant role in reducing the energy requirements for water splitting, it is equally important to integrate these catalysts onto solid supports for practical implementation.^7,8^ Homogeneous catalysts also present challenges related to catalyst recovery and long-term stability. The catalyst and products coexist in the same phase, so additional purification is needed to separate them for catalyst recycling, which complicates scaling up.^9^ Water oxidation requires a high potential; therefore, during this process, molecular WOCs may lose their ligands and degrade through oxidation, dimerization, or other mechanisms that deactivate catalytic activity.^10–12^ All of these limitations must be overcome for homogeneous catalysts to be employed in practical water-splitting applications. Heterogeneous catalysts have, therefore, been explored to overcome several of these limitations. When catalytic species are immobilized on electrode surfaces, direct electronic communication with the electrode eliminates diffusion limitations and allows more efficient utilization of active sites.^13^ In addition, heterogeneous catalysts can be readily separated from the reaction medium and reused, facilitating long-term operation and device integration. Transition metal oxide catalysts such as RuO_2_, IrO_2_, CoO_*x*_, and NiFeO_*x*_ have been identified as some of the top heterogeneous water oxidation catalysts because of their stability in harsh oxidative environments and high activity for water oxidation.^14–16^ However, there is typically no well-defined structural control of the catalytically active sites on these materials. The active sites are usually distributed throughout a complex surface containing crystallographic facets, defects, and amorphous domains, which makes it difficult to identify or systematically tune the catalytic environment.^17–19^ Unlike molecular catalysts, heterogeneous metal oxides generally provide limited opportunities for precise functionalization or electronic tuning, and structural heterogeneity can complicate mechanistic investigations and rational catalyst design.

MOFs offer a promising platform that bridges the gap between homogeneous and heterogeneous catalysis by combining the advantages of both systems. MOFs, also known as porous coordination polymers (PCPs), are a class of porous materials assembled from metal-containing nodes (secondary building units, or SBUs) and organic linkers.^20^ The composition and topology of MOF materials can be precisely controlled through careful selection of metal nodes, organic linkers, and reaction procedures.^21^ Significant research has been dedicated to investigating MOF-based catalysts for potential future applications in solar fuels, such as electrochemical CO_2_ reduction, hydrogen production, and oxygen reduction.^22–24^ MOFs present excellent platforms for heterogeneous WOCs for several key reasons. First, their large surface area provides a high density of reaction sites for the initiating catalytic water oxidation reactions. The sponge-like framework and interconnected pathways facilitate strong interactions between the bridging linkers and guest molecules while also allowing for rapid and effective molecular movement.^25^ Second, MOFs enable precise molecular-level integration with Ru-based WOCs, thanks to their highly ordered structure, which allows for the organized arrangement of different molecular components.^26^ Third, anchoring mononuclear Ru-based WOCs within MOFs prevents undesirable chemical interactions between catalyst molecules, simplifying the study of water oxidation reactions and potentially inhibiting catalyst degradation.^27,28^ Lastly, the easy separation of MOFs from solution facilitates the reuse of WOCs and the post-reaction characterization of the catalysts.^29^

In this work, we report the synthesis and electrochemical characterization of a ruthenium-based WOC [Ru­(Mebimpy)­(dcbpy)­H_2_O]^2+^ (Mebimpy = 2,6-bis­(1-methylbenzimidazol-2-yl)­pyridine, dcbpy = 5,5-dicarboxy-2,2′-bipyridine) incorporated into the UiO-67 framework, designated as RuM–UiO-67. The resulting RuM–UiO-67 thin films, grown directly on fluorine-doped tin oxide (FTO) substrates, exhibit robust water oxidation activity at near-neutral pH (pH 6) with an overpotential of ∼600 mV, a turnover frequency (TOF) of (0.32 ± 0.02) s^–1^ at 1.5 V vs NHE, and a Faradaic efficiency of 59%. Notably, the MOF structure enables a more than 12-fold enhancement in electroactive surface coverage relative to a monolayer of the same catalyst, underscoring the role of the framework in improving catalyst accessibility and charge transport. The work highlights the potential of MOF-based architectures for advancing molecular electrocatalysis and contributes to the design of next-generation artificial photosynthetic systems.

## Materials and Methods

### Chemicals

All chemicals and solvents were used as obtained without further purification, including RuCl_3_·3H_2_O (Ambeed, Inc. 97%), 2,2′-bipyridyl-5,5′-dicarboxylic acid (dcbpy, Ambeed, Inc. 97%), pyridine-2,6-dicarboxylic acid (Ambeed, Inc. 97%), *N*-methyl-1,2-benzenediamine dihydrochloride (Oakwood Chemicals, 97%), phosphoric acid 85% solution, lab grade (Lab Alley), 4,4′-biphenyldicarboxylic acid (BPDC, Oakwood Chemicals, 97%), zirconium­(IV) chloride (Sigma-Aldrich, 98%), deuterium oxide with 99.9 atom % D and 0.05 wt % 3-(trimethylsilyl)­propionic-2,2,3,3-d_4_ acid, sodium salt (Sigma-Aldrich), DMF (Fisher Scientific, HPLC grade, >99%), triethylamine (TEA, Sigma-Aldrich, 99%), acetonitrile (Fisher Scientific, HPLC grade), sodium hydroxide (Fisher Chemical), hydrochloric acid (36.5 to 38.0%, Fisher Chemical), lithium perchlorate (Sigma-Aldrich, >99%), lithium chloride (LiCl, Aldrich Chemical Company, Inc., >99%), ethanol (Fisher Scientific), acetone (Spectrum, HPLC grade), acetic acid (Oakwood Chemicals), and alconox detergent (Alconox, Inc.). FTO slides were acquired from Hartford Glass, Inc, USA.

### Nuclear Magnetic Resonance (NMR) Spectroscopy

All NMR experiments were performed by using a Bruker Avance NEO 400 NMR (400 MHz) Spectrometer. For MOF digestion samples, 1 M NaOH solution was added to 2–5 mg of particles suspended in D_2_O for 30 min in order to fully degrade the particles for quantitative assessment. For quantitative NMR, 64 scans and a relaxation delay of 10 s were used.

### Powder X-ray Diffraction (PXRD)

PXRD patterns for the MOF films were collected on a Bruker D8 Advance Wide-Angle X-ray diffractometer with a Cu kα (λ = 0.1541 nm) radiation source generated at 40 kV and 40 mA. The measurements were collected from 2θ values of 3° to 40° with a resolution of 0.02° at a rate of 0.25° per minute.

### Scanning Electron Microscopy (SEM)

The SEM images were collected with a Leo/Zeiss 1550 Schottky field-emission scanning electron microscope.

### UV–Vis Spectroscopy

Absorbance measurements were taken by using a Cary 5000 UV–Vis–NIR spectrometer controlled with Cary WinUV software. The scan application was used to collect spectra from 250 to 700 nm of the post-electrolysis solution from the water oxidation experiment.

### X-ray Photoelectron Spectroscopy (XPS)

XPS spectra were collected on a PHI 5000 Versa probe III spectrometer using an aluminum anode X-ray source with a photon energy of 1486.6 eV. Survey spectra were collected with a 25 W, 15 kV source producing a 100 μm beam with a scan range from 1100 to 0 eV, a step size of 0.5 eV, and a pass energy of 280 eV. Each elemental range except for the Ru 3p region was scanned for 15 sweeps, whereas the Ru 3p region was scanned for 450 and 900 sweeps for the pre- and post-electrolysis thin films, respectively. The spectra were fitted by using CasaXPS software. Charge correction was performed by using the adventitious C 1s peak at 284.5 eV.

### Electrochemical Measurements

Electrochemical measurements were conducted with a Pine Instruments WaveNow potentiostat using a three-electrode setup with the MOF film coated on an FTO slide as the working electrode, a Ag/AgCl (saturated KCl) reference electrode, and a platinum mesh counter electrode. All electrochemical experiments were done in 0.1 M LiClO_4_ in water electrolyte adjusted to pH = 6 prior to analysis with HCl. For cyclic voltammetry measurements, at least six sweeps were performed until the current response was stable, and the final cycle was reported. The scan rate was varied from 10 to 1000 mV/s to analyze the scan-rate dependence of the redox process and evaluate charge-transport behavior within the MOF film. Bulk electrolysis experiments were performed in a three-compartment electrochemical cell, with each compartment separated by porous glass frits. The solution was purged with argon for at least 30 min before each electrolysis experiment. The dissolved oxygen concentration was detected with a Unisense OX-NP oxygen sensor.

### Inductively Coupled Plasma Mass Spectrometry (ICP–MS)

For post-electrolysis analysis, 1 mL of the electrolyte solution was combined with 2 mL of 70% HNO_3_ and heated at 90 °C for 1 h. The mixture was then diluted with water to achieve a final HNO_3_ concentration of 6.7% (v/v). The ruthenium and zirconium contents were measured by using an Agilent 7900 inductively coupled plasma mass spectrometer.

### Brunauer–Emmett–Teller (BET) Surface Area

N_2_ physisorption measurements were performed by using an Anton Paar Autosorb iQ C-XR surface area analyzer to determine the BET surface area. Prior to analysis, 100 mg of the samples were activated under dynamic vacuum at 100 °C for 12 h to remove residual solvents and guest molecules. Nitrogen adsorption–desorption isotherms were collected at 77 K over a relative pressure (*P*/*P*_0_) range of approximately 10–6 to 0.995. The BET surface area was calculated by fitting the adsorption data to the linear region of the BET plot.

## Results and Discussion

### Synthesis and Characterization

Previous mechanistic studies of the molecular complex [Ru­(Mebimpy)­(dcbpy)­H_2_O]^2+^ as a homogeneous water oxidation catalyst provide an important foundation for understanding its behavior when incorporated into heterogeneous assemblies such as MOFs.^30^ [Ru­(Mebimpy)­(dcbpy)­H_2_O]^2+^ (Figure 1a) was synthesized using established processes and incorporated into the UiO-67 MOF backbone.^30^ RuM–UiO-67 was synthesized using a modified approach that was previously employed for other UiO analogs.^31^ A clean FTO was put in a combination of [Ru­(Mebimpy)­(dcbpy)­H_2_O]^2+^, biphenyl-4,4′-dicarboxylic acid (BPDC), and ZrCl_4_ in DMF, which was heated at 120 °C for 1 day. [Ru­(Mebimpy)­(dcbpy)­H_2_O]^2+^ and BPDC link with Zr^4+^ ions to create oxo clusters (Figure 1b), which then form the characteristic pseudo-octahedral UiO-type structure (Figure 1c). To remove unreacted precursors and loosely bound species, the thin films were soaked in DMF and acetonitrile for 3 days with new solvent added each day and then evacuated at room temperature for further use.

**Figure 1:**
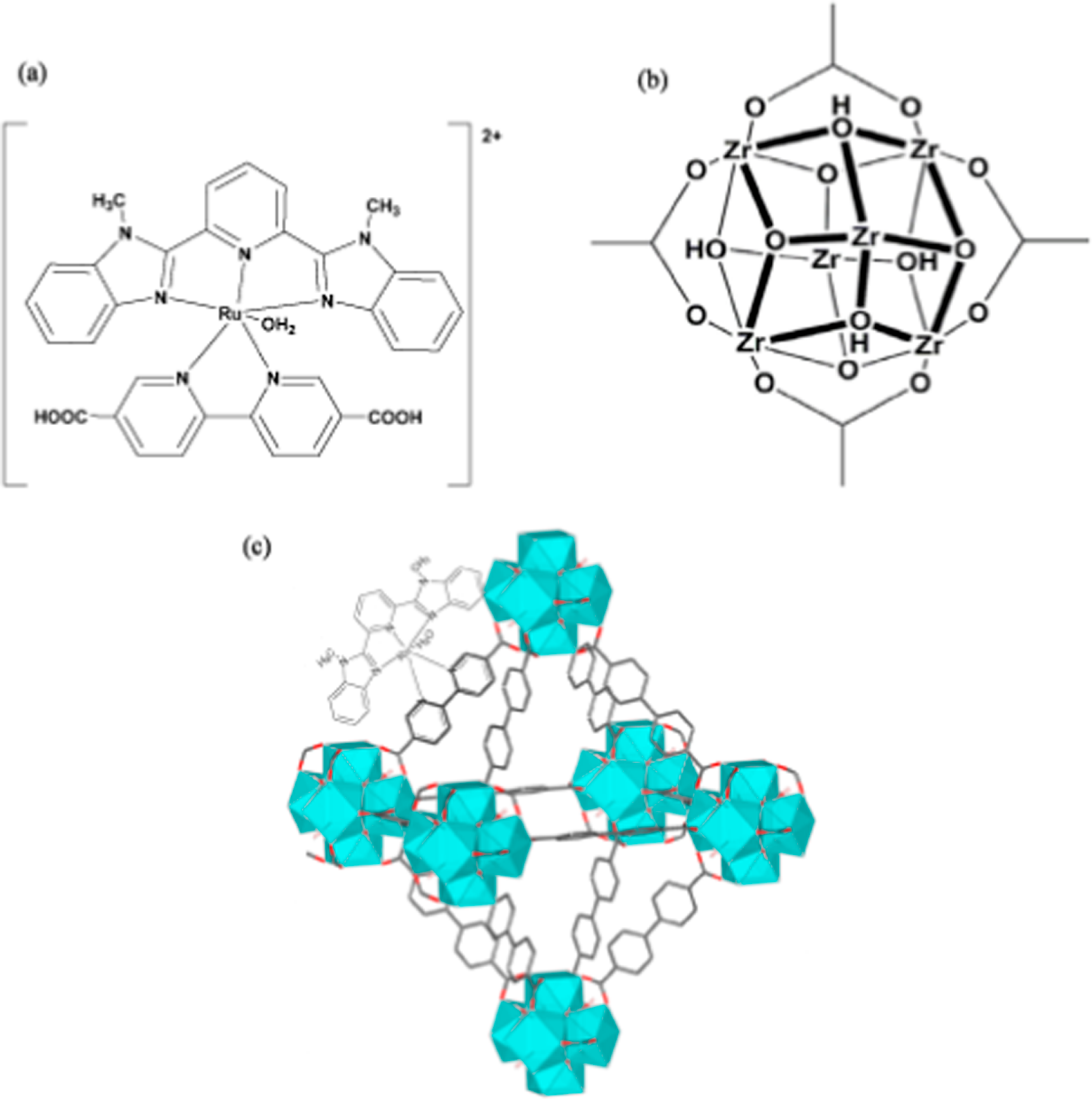
Structures of (a) [Ru­(Mebimpy)­(dcbpy)­H_2_O]^2+^ molecular catalyst, (b) Zr_6_O_4_(OH)_4_(COO)_12_ cluster of the UiO-67 MOF, and (c) catalyst-incorporated RuM-UiO-67 MOF.

Powder X-ray diffraction (PXRD) patterns of the thin film showed a close match to the simulated diffraction pattern of pristine UiO-67, confirming that the incorporation of the ruthenium catalyst into the MOF did not disrupt its overall crystallinity or topology (Figure 2a). Structural integrity is essential for maintaining the porosity and accessibility of catalytic sites throughout the film. Scanning electron microscopy (SEM) analysis (Figure 2b) revealed that the RuM–UiO-67 thin films consist of densely intergrown, pseudo-octahedral crystallites, a morphology characteristic of bulk UiO-67.^32,33^ The particle size distribution likely arises from the absence of acid modulators, such as acetic or benzoic acid, which are often used to control nucleation and crystal growth in MOF synthesis, yet the particles adhered well to the FTO substrate and formed a continuous film.^34,35^

**Figure 2:**
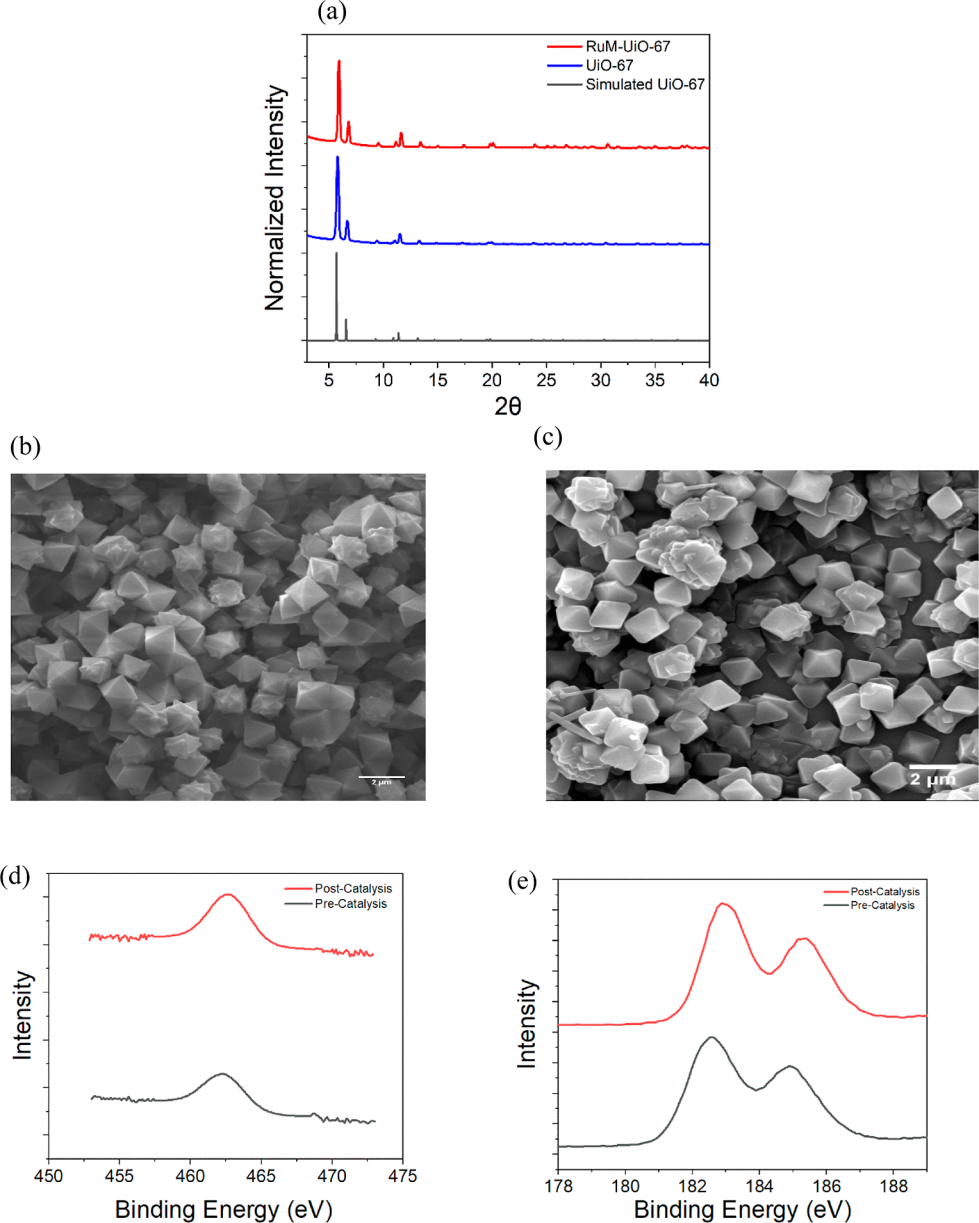
(a) PXRD pattern of synthesized RuM–UiO-67 and UiO-67 MOFs as compared to a simulated pattern for UiO-67 (CCDC 1021002). SEM images of the RuM–UiO-67 thin film before (b) and after (c) electrocatalysis. XPS spectra of Ru 3p (d) and Zr 3d peaks (e) before and after electrocatalysis.

X-ray photoelectron spectroscopy (XPS) further confirmed the successful incorporation of the ruthenium complex. The presence of a distinct Ru 3p_3/2_ peak at a binding energy (BE) of 462.43 eV, along with the characteristic Zr 3d_5/2_ and 3d_3/2_ doublet at 182.55 and 184.92 eV, respectively, indicates that both the Zr-based MOF nodes and the Ru centers coexist in the film (Figure 2d,e). ^1^H NMR analysis of the digested RuM–UiO-67 MOF sample confirmed the successful incorporation of the catalyst into the UiO-67 framework. Integration of the characteristic aromatic signals from the RuM catalyst and the BPDC linker revealed an average ratio of ∼1:5, which corresponds to approximately one RuM catalyst per Zr_6_ node in the structure (Figure S1). The relatively low loading reflects the steric demand of the RuM ligand, which restricts the number of functionalized linkers that can be accommodated within the UiO-67. The film thickness (∼2 μm) was measured using cross-section SEM imaging (Figure S2). Surface area measurements obtained from N_2_ sorption at 77 K confirm that RuM catalyst incorporation preserves the high porosity characteristic of the UiO-67 framework (Figure 3a,b). The BET surface area of RuM–UiO-67 was measured to be ∼1200 m^2^/g, which is lower than that of pristine UiO-67 but consistent with the expected reduction upon linker functionalization and catalyst incorporation.^36^ Similar decreases in accessible surface area have been reported for other Ru-modified UiO-67 materials following introduction of bulky terpyridine-type ligands.^37^

**Figure 3:**
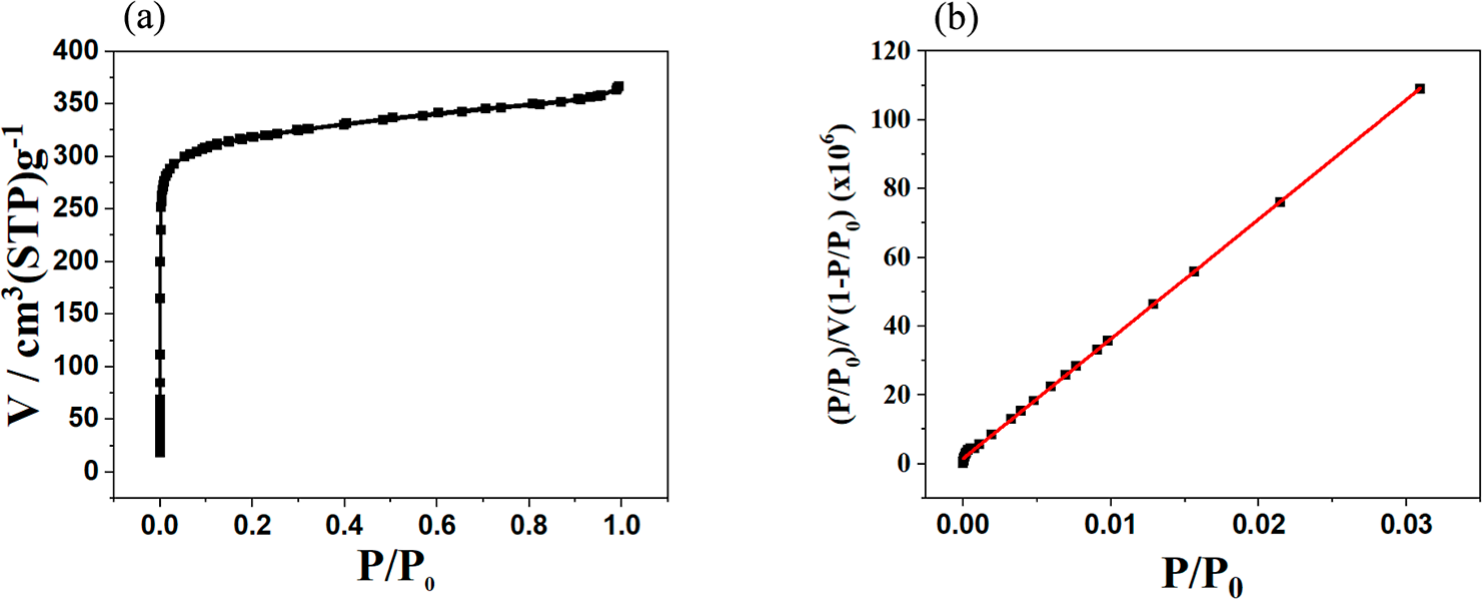
(a) Nitrogen adsorption isotherm and (b) BET surface area plot for RuM–UiO-67.

### Electrochemical Characterization

The electrochemical and catalytic behavior of related mononuclear ruthenium polypyridyl aqua complexes has been previously reported.^38,39^ In particular, Concepcion and co-workers demonstrated that complexes of the type [Ru­(LLL)­(LL)­(H_2_O)]^2+^ (where LLL is a tridentate ligand like terpyridine and LL is a bidentate ligand like bipyridine) can function as single-site water oxidation catalysts, where ligand variations strongly influence redox potentials and catalytic activity.^30^ Electrochemical studies revealed sequential proton-coupled electron transfer (PCET) processes, leading to high-valent ruthenium oxo intermediates, which participate in the formation of the O–O bond through the nucleophilic attack of water. Catalytic water oxidation was observed both electrochemically and in chemical oxidation experiments using Ce^4+^, confirming that these molecular complexes can promote oxygen evolution through well-defined molecular mechanisms. These studies established important structure–reactivity relationships for Ru polypyridyl water-oxidation catalysts and provided a useful reference point for evaluating the catalyst’s behavior after immobilization within the MOF.

The electrochemical properties of thin films and the corresponding molecular catalyst under the same conditions were examined by using linear sweep voltammetry (LSV) and cyclic voltammetry (CV). The linear sweep voltammograms (Figure 4a) of RuM–UiO-67 and the 1 mM RuM catalyst in solution reveal distinct redox behavior. The Ru-based redox peak in the MOF thin film appears at a more positive potential (∼0.84 vs ∼0.75 V) relative to the solution-phase catalyst. The anodic shift suggests that incorporation of the RuM unit into the UiO-67 framework renders the Ru center less electron-rich, likely due to coordination to the MOF environment and restricted solvation. These interactions increase the energy required for oxidation, which is consistent with the observed potential shift. Such behavior reflects the electronic influence of the framework and highlights the role of immobilization in tuning redox properties of molecular catalysts as well as a potential contribution owing to the functionalization of carboxylic acid groups at the 5,5′-positions.^40^

**Figure 4:**
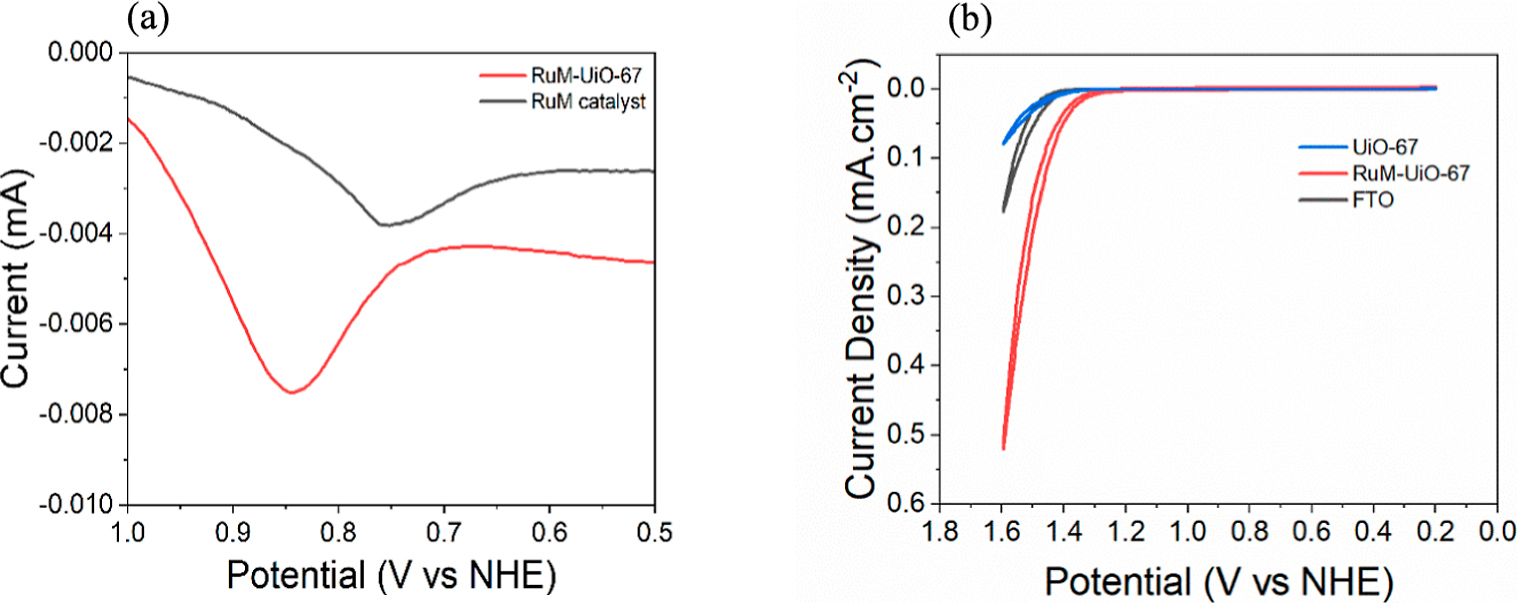
(a) LSV of the RuM–UiO-67 thin film (red) and 1 mM RuM catalyst (black) solution in 0.1 M aqueous LiClO_4_. (b) CVs of FTO (black), UiO-67 (blue), and RuM–UiO-67 thin film (red) in 0.1 M aqueous LiClO_4_ (pH 6) at a scan rate of 100 mV/s.

When the applied potential exceeded 1.4 V versus NHE in 0.1 M LiClO_4_ at pH 6, the RuM–UiO-67 thin film exhibited a noticeable increase in current, suggesting the onset of catalytic activity (Figure 4b).^41^ The enhanced response is attributed to the presence of ruthenium WOC, which is proposed to serve as the active species driving the reaction. The catalytic current observed for the RuM–UiO-67 film was significantly higher than that of both bare FTO and the catalyst-free UiO-67 thin film. Interestingly, the UiO-67 film without any catalyst showed even lower current than bare FTO, likely due to partial blockage of the electrode surface by the MOF, which reduces the number of accessible catalytic sites.^42^

A critical factor in the design and performance of electrocatalytic MOFs is their ability to facilitate efficient charge transport, as both electron and proton mobility within the framework directly impact catalytic efficiency.^43^ Given that the UiO series of MOFs are generally not inherently conductive, understanding the origin of the electrochemical current observed in experiments is essential. Two main scenarios can explain the current response. In the first, only redox-active sites located near the electrode interface participate in electron transfer, while the majority of the MOF film remains electrochemically inactive. In the second, charge transport occurs through a redox hopping mechanism, where electrons migrate between redox centers, allowing the redox process to propagate deeper into the film and generate a diffusion-controlled current response. Redox hopping is a charge transfer mechanism that involves the movement of electrons and counterbalancing ions through self-exchange reactions between redox-active centers.^44^ In the context of MOFs, redox hopping involves electron transfer between redox-active metal centers and ligands within the framework. The latter mechanism has been previously demonstrated by Morris et al. through primarily potential jump experiments.^45^ To distinguish between these possibilities in the current system, CV was conducted on RuM–UiO-67 thin films across a wide range of scan rates (10 to 1000 mV/s). The scan-rate-dependent behavior provides insights into the charge-transport dynamics and the extent to which electron hopping or surface-confined redox processes dominate within the MOF film.

For diffusion-controlled redox processes, the peak current typically adheres to the Randles–Sevcik equation (eqs 1 and 2):

1 $$i_{p}=0.4463nFAC{\left(nFvD/RT\right)}^{1/2}$$

2 $$\log\left(i_{p}\right)=0.5\;\log\left(v\right)+\log\left[0.4463nFAC{\left(nFD/RT\right)}^{1/2}\right]$$

Conversely, if *i*_p_ arises from surface-bound redox species, it should follow eqs 3 and 4:^46^

3 $$i_{p}=n^{2}F^{2}vA\Gamma /4RT$$

4 $$\log\left(i_{p}\right)=\log\left(v\right)+\log\left[n^{2}F^{2}vA\Gamma /4RT\right]$$

where Γ is the amount of active species adsorbed on the electrode, *n* is the number of electrons transferred, *F* is the Faraday constant, *A* is the electrode surface area, *C* is the concentration of the redox-active species, *v* is the scan rate, *D* is the diffusion coefficient, *R* is the molar gas constant, and *T* is the absolute temperature.

The cyclic voltammograms (Figure 5a) show a pair of well-defined anodic and cathodic peaks centered around ∼0.84 V vs NHE, with the peak currents increasing proportionally with scan rate. The redox couple displays near-symmetric peak shapes with minimal change in peak separation at varying scan rates, suggesting a reversible redox behavior with fast electron transfer kinetics. To further evaluate the nature of the process, we plotted the logarithmic values of the anodic peak currents of the Ru^2+^/Ru^3+^ redox couple versus logarithm of the scan rate (Figure 5b) which exhibits a slope of 0.88. This value is close to the theoretical value of 1 (eq 4) expected for a surface-confined redox process and indicates that the electrochemical response is predominantly controlled by surface-bound Ru sites within the MOF film.^47^ Additionally, the peak current was plotted against the scan rate, yielding a linear relationship with an excellent correlation coefficient (*R* ^2^ = 0.99), as shown in Figure S3 which is in accordance with eq 3 for a reversible surface-confined charge transfer process.^48^ The fraction of electrochemically accessible Ru sites within the RuM–UiO-67 MOF thin film was estimated from integration of the Ru^3+/2+^ redox feature in the CV. The integrated charge was converted to the number of electroactive centers using Faraday’s law. The results indicate that (43.2 ± 1.5)% of the incorporated Ru centers are electrochemically active, suggesting that only a surface-accessible or percolating subset of Ru centers participates in redox hopping, while the rest remain electrochemically isolated (Figure S4).

**Figure 5:**
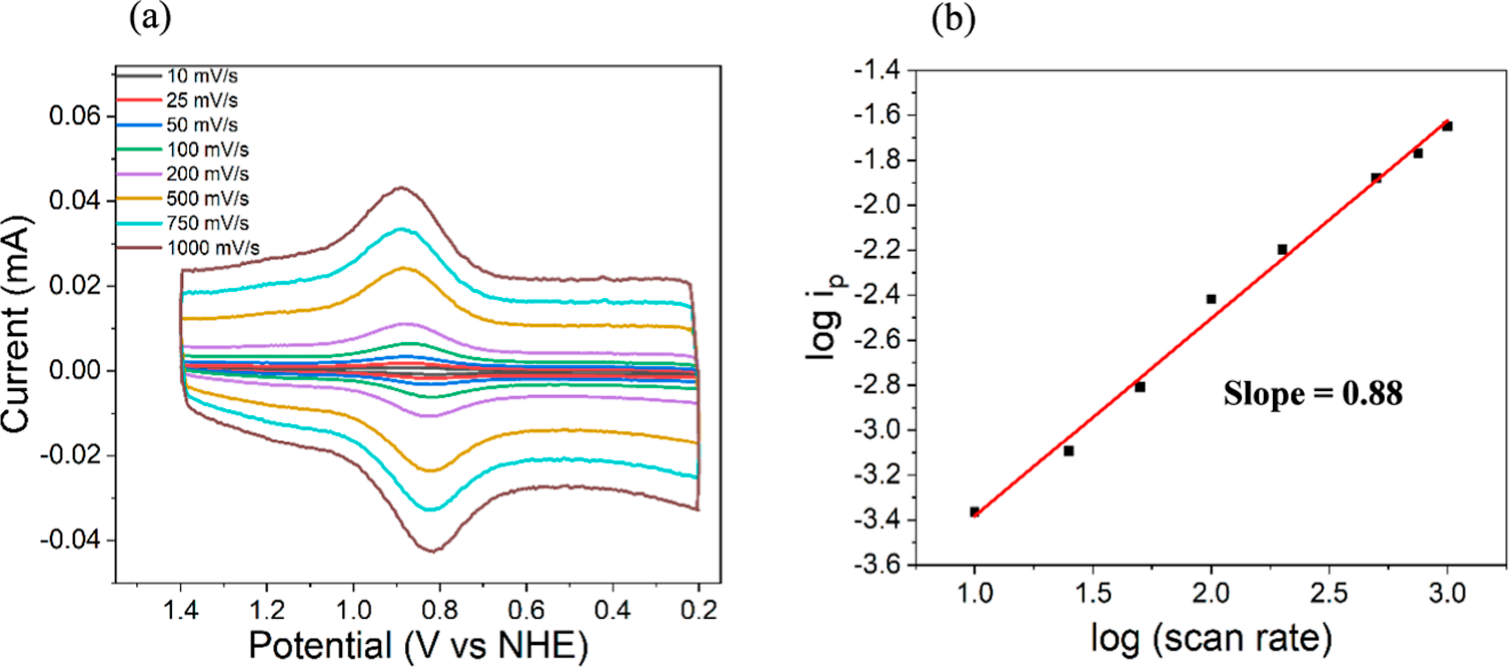
(a) CV of the RuM–UiO-67 thin film at scan rates from 10 to 1000 mV/s in 0.1 M aqueous LiClO_4_. (b) The log­(*i*_p_) versus log­(scan rate) plot.

In the quantification of charge transport within MOFs, the apparent diffusion coefficient (*D*_app_) serves as a crucial metric for evaluating overall charge mobility. *D*_app_ reflects two coupled processes: electron self-exchange between redox-active sites and the diffusion of counterions that maintain charge neutrality. Techniques such as chronoamperometry, spectroelectrochemistry, and cyclic voltammetry are widely employed to measure *D*_app_.^49^ Chronoamperometry was employed here to determine the *D*_app_. The technique models time-dependent current behavior following a potential step using the Cottrell equation (eq 5):

5 $$i=\frac{nFAC^{0}\sqrt{D_{app}}}{\sqrt{\pi t}}$$

where *i* is the current (*A*), *n* is the number of electrons transferred, *F* is the Faraday constant (C/mol), *C* ^0^ is the concentration of the redox-active species (mol/cm^3^), and *t* is the time (s). A plot of current versus 1/*t* ^1/2^ enables the extraction of *D*_app_. Chronoamperometric measurements were performed on the MOF films by applying a potential of 800 mV vs Ag/AgCl to drive full oxidation of the redox-active centers. The resulting current response and Cottrell plot are shown in Figure 6.

**Figure 6:**
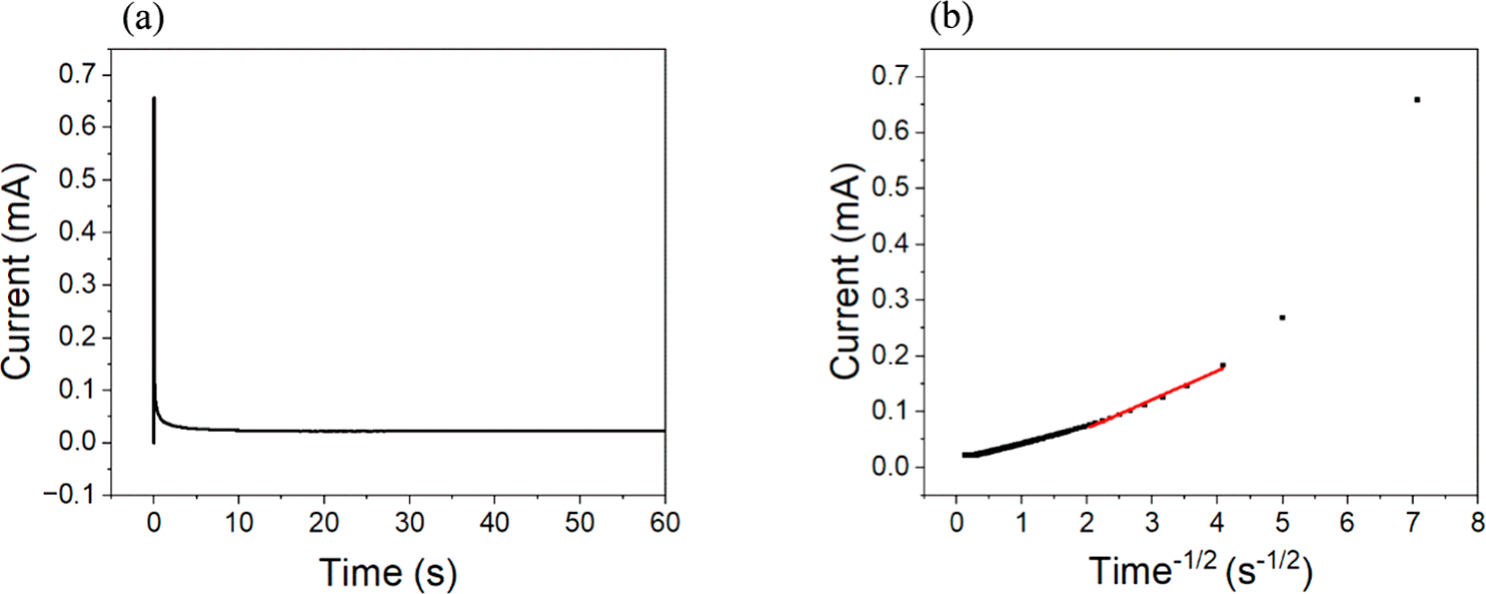
(a) Chronoamperometry data for RuM–UiO-67. (b) Corresponding Cottrell plot for RuM–UiO-67. The red line indicates the linear region used to determine the slope.

The apparent diffusion coefficient for all films studied was calculated to be (5 ± 1.3)×10^–11^ cm^2^/s based on the approach. The measured *D*_app_ (∼10^–11^ cm^2^/s) falls within the broad range reported for redox-active MOFs (10^–12^–10^–9^ cm^2^/s) and is consistent with charge transport mediated by electron hopping and counterion diffusion within densely functionalized UiO-type frameworks (Table S1). The *D*_app_ of RuM–UiO-67 reflects coupled charge transport in redox-active MOFs, governed by both electron self-exchange between neighboring Ru centers and counterion diffusion through the framework.^31^ Direct measurement of electron self-exchange rates for Ru-based molecular catalysts is experimentally challenging, and hence, *D*_app_ is typically treated as an effective parameter incorporating ion transport within the pores.^50^ In RuM–UiO-67, incorporation of the bulky RuM catalyst reduces the BET surface area relative to that of pristine UiO-67, indicating partial pore occupation that is expected to impede ion mobility and slow overall charge propagation. Such reduced charge-transport rates are commonly observed upon incorporation of bulky molecular catalysts and increased linker functionalization.^26,51^ The electroactive-site coverage of the film reported here is over 12 times greater than that of a fully packed monolayer of [RuM–OH_2_ ]^2+^ on the same FTO electrode surface (Figure S4).^52^ The enhanced electroactive-site coverage arises from the volumetric incorporation of redox centers within the MOF architecture.

### Water-Oxidation Electrolysis

We performed a water concentration-dependent study with a RuM–UiO-67 thin film to check its water oxidation reactivity (Figure S5). Increased water concentration resulted in increased catalytic current starting from a non-aqueous electrolyte solution of 0.1 M LiClO_4_ in CH_3_CN. The current monitored at 1.5 V vs NHE varied linearly with [H_2_O], which indicates that the reaction is second order with respect to water oxidation mediated by the RuM–UiO-67 thin film.^53^

Controlled potential electrolysis (CPE) was carried out on RuM–UiO-67 thin films to evaluate their effectiveness in driving electrochemical water oxidation reactions (WOR), as shown in Figure 7a. CPE serves as a critical tool for evaluating the operational robustness of electrocatalysts, enabling the direct observation of performance over extended periods and under constant potential conditions. The electrolysis process was conducted for 1 h in an electrolyte composed of 0.1 M LiClO_4_ at pH 6. For comparison, control tests using catalyst-free UiO-67 thin films and bare FTO were performed under identical conditions. When a potential of 1.5 V versus NHE (corresponding to an overpotential of ∼600 mV) was applied, the RuM–UiO-67 thin films exhibited a maximum sustained catalytic current of 7.6 μA/cm^2^.

**Figure 7:**
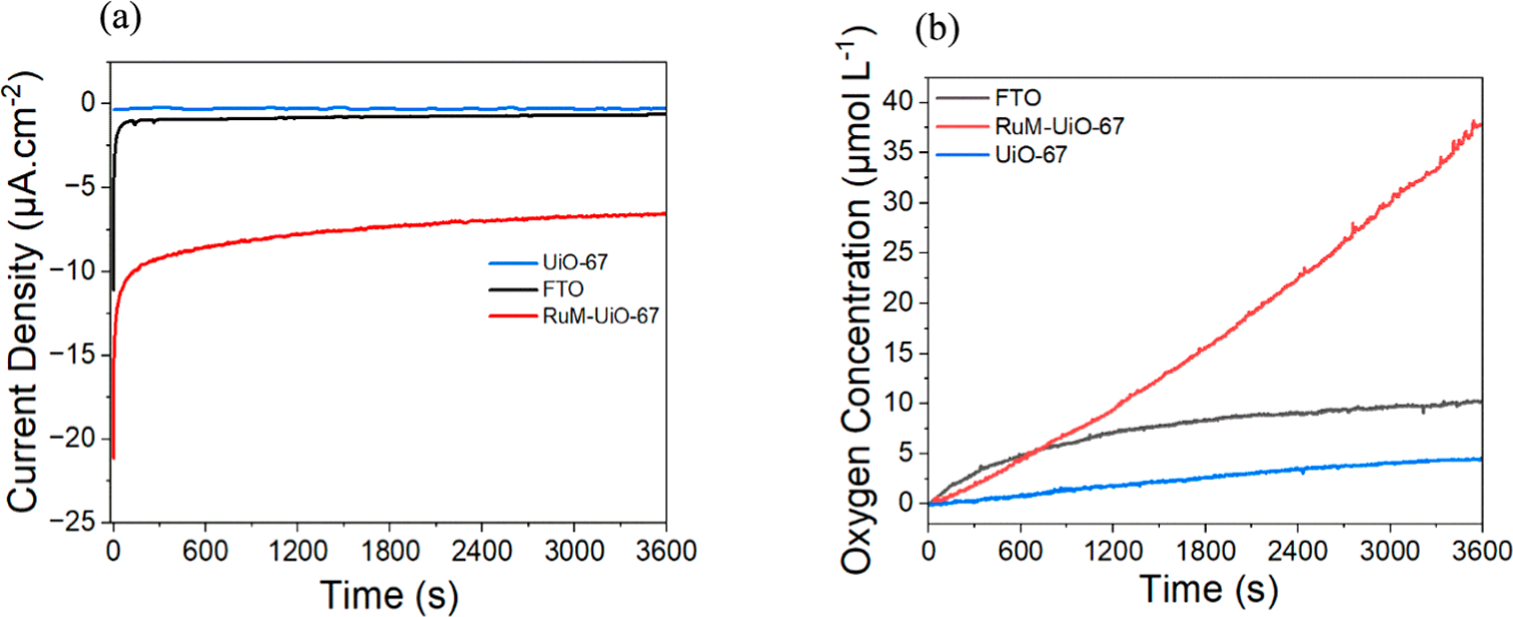
(a) CPE profiles for the RuM–UiO-67 thin film, catalyst-free UiO-67, and bare FTO, performed at a fixed potential of 1.5 V vs. NHE for 1 h in a 0.1 M aqueous LiClO_4_ at pH 6. (b) Corresponding oxygen evolution data collected during electrolysis for each electrode.

During the CPE experiments, the RuM–UiO-67 thin film generated (0.86 ± 0.21) μmol of O_2_, in contrast to (0.15 ± 0.10) μmol for the catalyst-free UiO-67 thin film and (0.31 ± 0.02) μ mol for the bare FTO electrode (Figure 7b). These findings align with the cyclic voltammetry data, which suggested that the UiO-67 framework limits access of reactive species to the underlying conductive surface, leading to reduced catalytic activity. Notably, similar physical occlusion of the FTO surface is expected in the RuM–UiO-67 sample, yet it still delivers significantly higher O_2_ output, indicating that the enhanced activity is attributable to the incorporation of the Ru-based WOC. Therefore, the most meaningful comparison is between the RuM–UiO-67 and the undoped UiO-67 films, where a roughly six-fold increase in oxygen evolution is observed upon Ru catalyst incorporation. The performance enhancement is further corroborated by Faradaic efficiency measurements, which were calculated to be (59 ± 2)% for RuM–UiO-67, (40 ± 0.7)% for FTO, and (20 ± 4)% for UiO-67. These results collectively emphasize the critical role of the Ru catalyst in promoting the efficient electrochemical water oxidation. The Faradaic efficiency below 100% may arise from processes other than O_2_ evolution, like partial two-electron oxidation to H_2_O_2_. To further probe competing pathways, a qualitative KI/acetic acid test was performed on the post-electrolysis solution, and a persistent yellow coloration was observed, consistent with the formation of H_2_O_2_ (Figure S6). Iodometric spectrophotometry of the post-electrolysis electrolyte revealed the quantitative amounts of H_2_O_2_ during water oxidation (Figure S7). A 3.5 mL aliquot of the reaction solution contained ∼31.6 μM H_2_O_2_, corresponding to 1.106 μmol of peroxide in the electrolyte. The quantity is comparable to the amount of evolved O_2_ under identical conditions, indicating that a substantial portion of the oxidizing equivalents is diverted through the competing two-electron pathway to H_2_O_2_. The detection of H_2_O_2_ therefore accounts for the Faradaic efficiency loss observed for the RuM–UiO-67 film and highlights peroxide formation as an important side reaction in MOF-supported molecular water-oxidation catalysts.^54,55^ Furthermore, the catalyst-free UiO-67 film produced ∼11.68 μmol of H_2_O_2_ under identical conditions. These values correspond to an O_2_/H_2_O_2_ ratio of 0.78 for RuM–UiO-67 compared to 0.013 for UiO-67 alone. Incorporation of the Ru catalyst therefore increases O_2_ production by approximately 5.7-fold, indicating that the molecular Ru centers promote the four-electron oxygen evolution pathway, while the bare MOF predominantly follows the two-electron peroxide formation pathway. The RuM–UiO-67 thin films were evaluated for repeated water-oxidation activity and exhibited consistent performance across multiple electrolysis cycles, each using a newly prepared electrolyte (Figure S8). To evaluate the effect of MOF immobilization on catalyst stability, the chemical water oxidation performance of RuM–UiO-67 was directly compared with that of the corresponding homogeneous RuM catalyst under identical conditions using cerium­(IV) ammonium nitrate (CAN) as a sacrificial oxidant in acidic media (pH 0.5). In a typical experiment, the amount of the dissolved RuM catalyst was matched to the Ru loading in the MOF, and oxygen evolution was monitored using an oxygen probe under an inert atmosphere. The time-dependent oxygen production revealed that the homogeneous RuM catalyst exhibited a lower initial rate of oxygen evolution and underwent rapid deactivation, with O_2_ production ceasing after approximately 15.6 min. In contrast, the RuM–UiO-67 material sustained oxygen evolution for over 40 min under the same conditions, indicating significantly enhanced catalytic durability upon immobilization within the framework (Figure S9). This improved stability is attributed to the confinement of the molecular catalyst within the MOF structure, which mitigates common deactivation pathways that are prevalent in homogeneous systems.

Since not all Ru-based WOCs embedded within the MOF thin film are electrochemically active, the TOF was calculated based on the estimated electroactive-site coverage. Using the approach, the TOF for the RuM–UiO-67 thin film was determined to be approximately (0.32 ± 0.02) s^–1^, reflecting the intrinsic activity of the accessible Ru catalytic centers. For comparison, the similar homogeneous [Ru­(Mebimpy)­(bpy)­(OH_2_)]^2+^ catalyst shows a TOF of ∼0.028 s^–1^ under Ce­(IV) oxidation, indicating that the MOF-based system shows over an order of magnitude greater catalytic activity under milder, electrocatalytic conditions.^19^ To further probe the kinetics of the electrocatalytic process, Tafel analysis (Figure S10) was conducted, yielding a Tafel slope of (124 ± 2) mV/dec. The observed Tafel slope of 124 mV/dec indicates that the rate-determining step corresponds to the first electron transfer, which, in this heterogenized framework, likely reflects sluggish charge injection from the electrode through the MOF backbone to the ruthenium centers.^56–58^ This electronic bottleneck is further supported by the limited electrochemical accessibility (∼43%), suggesting that only surface-proximal Ru sites are catalytically active due to intrinsically slow charge transport within the MOF. Despite these electronic limitations, the chemical kinetics at the accessible Ru sites display a second-order dependence on water concentration, consistent with the Atom–Proton Transfer (APT) model proposed by Chen et al.^59^ In this model, the formation of an O–O bond proceeds via a water nucleophilic attack (WNA) on a high-valent [Ru^V^ = O]^3+^ intermediate, with one water molecule acting as the nucleophile and a second serving as a proton-accepting base to stabilize the transition state (Figure S11). The spatial isolation of the Ru centers within the MOF pores likely enforces this single-site WNA pathway. The coexistence of O_2_ and H_2_O_2_ can be explained by kinetic branching at the [Ru^III^–OOH]^2+^ intermediate.^60^ Under normal 4-electron process, this species is rapidly oxidized to release O_2_, but the slow charge transport delays delivery of the third and fourth electrons, allowing partial hydrolytic release as H_2_O_2_.

### Post-Catalysis Stability Characterization

To evaluate the structural and chemical stability of the RuM–UiO-67 thin film under catalytic conditions, a comprehensive post-electrolysis characterization was performed. PXRD patterns collected before and after catalysis also showed no significant differences, confirming that the crystalline structure of the UiO-67 framework remained intact throughout the experiment (Figure S12). SEM imaging revealed no appreciable morphological changes in the MOF film following 1 h of electrolysis, indicating that the overall crystal integrity and particle architecture were retained (Figure 2b). To probe potential changes in the oxidation states or coordination environments of the metal centers, XPS was conducted on the films before and after catalysis. The binding energy of the Ru 3p_3/2_ peak was 462.43 eV before catalysis and 462.45 eV afterward, indicating that the ruthenium centers remained chemically stable under the applied electrochemical conditions. Prior to catalysis, the Zr 3d_5/2_ peak was observed at 182.60 eV with a full width at half-maximum (FWHM) of 1.74 eV, while after catalysis, the peak appeared at 182.98 eV with an FWHM of 1.60 eV. Similarly, the Zr 3d_3/2_ peaks shifted from 184.94 to 185.34 eV post catalysis (Figure 2d,e). These minimal shifts suggest a surface-level electronic modification with no significant changes in the oxidation state or coordination for either Ru or Zr during the catalytic process. This interpretation is firmly supported by the PXRD patterns and SEM images, which remain the same before and after catalysis, confirming that the long-range crystalline order and overall morphology of the framework remain unchanged.

To assess potential catalyst leaching, electronic absorption spectroscopy was performed on the post-electrolysis electrolyte solution. The absence of any detectable absorption features in the spectral range of the Ru complex indicates that no intact catalyst was released into solution under the applied conditions (Figure S13). Further ICP analysis revealed that only about 3.44% of the total ruthenium content was released into the solution. Together, these observations strongly suggest that the majority of Ru centers remained bound within the MOF during catalysis and the observed activity originates from the intact RuM–UiO-67 film rather than dissolved species. Although minor particle detachment from the FTO substrate cannot be fully ruled out, the preserved morphology and unchanged spectroscopic signatures point to excellent film stability. These findings support the conclusion that the RuM–UiO-67 architecture remains structurally and chemically robust under electrocatalytic water oxidation conditions at an operating pH.

## Conclusions

In this study, we report the successful incorporation of a benzimidazole-based ruthenium water oxidation catalyst into the UiO-67 MOF, forming thin films directly grown on FTO substrates. The incorporated Ru WOC retained its electrochemical activity within the MOF environment, displaying a low overpotential of ∼600 mV for water oxidation under near-neutral pH conditions. At an applied potential of 1.50 V vs NHE, the system achieved a TOF of (0.32 ± 0.02) s^–1^ and a Faradaic efficiency of ∼59% for oxygen evolution. The enhanced scan-rate dependence and redox behavior revealed a polydisperse distribution of electroactive sites throughout the film, with a 12-fold increase in surface coverage compared to a monolayer of the same catalyst on bare FTO. Compared with the molecular version, the hybrid RuM–UiO-67 MOF extended the lifespan of its active catalytic components and showed better recyclability. Post-catalysis characterization, including SEM, PXRD, XPS, and electronic absorption spectroscopy of the electrolyte, confirmed that the MOF structure and metal centers remained chemically and structurally stable throughout electrolysis, with no detectable leaching of intact Ru species into solution. These findings highlight the advantages of the MOF-based architecture for the stabilization and spatial distribution of molecular catalysts, enabling enhanced performance under mild aqueous conditions. The robust activity and stability demonstrated by the RuM–UiO-67 system underscore its potential as a platform for the development of next-generation, molecularly tunable, and device-integrated electrocatalysts for water splitting applications.

## Supplementary Material



## Notes

Synthesis of the ligand and water oxidation catalyst; experimental procedures for MOF synthesis; ^1^H NMR spectrum of digested RuM–UiO-67; cross-section SEM image of the RuM–UiO-67 MOF thin film on FTO; peak current versus scan rate plot; electroactive surface area comparison of RuM–UiO-67: MOF vs monolayer; relationship between the catalytic current of RuM–UiO-67 vs [H_2_O] in 0.1 M LiClO_4_/CH_3_CN; KI–acetic acid aqueous solution photographed before and after addition of an aliquot of the electrolyte; UV–vis spectra of increasing concentrations of standard H_2_O_2_ solutions and postcatalysis solution; repeated water-oxidation electrolysis of RuM–UiO-67 thin films; oxygen evolution data for RuM–UiO-67 and RuM catalyst; Tafel plot of RuM–UiO-67 in 0.1 M aqueous LiClO_4_; probable mechanistic pathways for competitive water oxidation in RuM–UiO-67 MOF; PXRD patterns of a RuM–UiO-67 film pre- and postcatalysis; and UV–vis absorption spectra of 0.01 M molecular [Ru­(Mebimpy)­(dcbpy)­H_2_O]^2+^ catalyst­(PDF)
