# Supplementary material for: Functionalized Metal–Organic Framework Thin Films for Stable and Efficient Electrochemical Water Oxidation under Near-Neutral Conditions
Source: ACS Electrochem. 2026 Apr 15;2(5):1282–92. doi: 10.1021/acselectrochem.6c00031 (PMC13158918; doi:10.1021/acselectrochem.6c00031)
Supplement: Supplementary file 1 [file ec6c00031_si_001.pdf]

Supporting Information

for

Functionalized Metal–Organic Framework Thin Film for Stable and Efficient Electrochemical Water Oxidation under Near-Neutral Conditions

Sumanta Basak [a], Arshia Sulaiman [a], and Amanda J. Morris [a,b*]

[a]Department of Chemistry, Virginia Polytechnic Institute and State University, Blacksburg, Virginia 24061, United States

[b]Macromolecules Innovation Institute, Virginia Polytechnic Institute and State University, Blacksburg, Virginia 24061, United States

*Corresponding author: ajmorris@vt.edu

**Contents**	**Page**
Synthesis of ligand and [Ru(Mebimpy)(dcbpy)H_2O]$^{2+}$	**S2-S3**
Fabrication of RuM-UiO-67 Thin Film and SEM Cross-section	**S3-S4**
Peak current versus scan rate plot	**S5**
Electroactive Surface Area Comparison of RuM-UiO-67: MOF vs Monolayer	**S5**
The relationship between of catalytic current of RuM-UiO-67 vs [H_2O]	**S6**
KI/acetic-acid colorimetric test and Iodometric spectrophotometry	**S7**
Repeated water-oxidation of RuM-UiO-67 thin films and Oxygen Evolution	**S9**
Tafel plot and Post-Catalysis Characterizations	**S9-S10**
Diffusion coefficients for MOFs and comparison with the RuM–UiO-67	**S11**

1. Synthesis of the Ligand and Catalyst

1.1 Synthesis of 2,6-bis(1-methylbenzimidazol-2-yl)pyridine (Mebimpy)

To prepare the catalyst, we first prepared the ligand, 2,6-bis(1-methylbenzimidazol-2-yl)pyridine (Mebimpy), by a modification of the procedure reported by Xu et al.[1] Briefly, Pyridine-2,6-dicarboxylic acid (3.35 g, 20 mmol) and N-methyl-1,2-phenylenediamine (5.38 g, 44 mmol) were stirred in 40 mL of 85% phosphoric acid at ~230 °C for 4 hours. The dark green reaction mixture was then poured into 1 L of cold water with vigorous stirring. After cooling to room temperature, the resulting blue solid was filtered, washed, and treated with hot 10% aqueous sodium carbonate (300 mL). The solid was collected, recrystallized from methanol, and obtained as a white powder (82% yield). ^{1}H NMR (400 MHz, $CDCl_3$) δ 8.43 (ddd, 2H), 8.10 – 8.01 (m, 1H), 7.88 (dtd, 2H), 7.46 (d, 2H), 7.38 (ddt, 4H), 4.26 (s, 6H).

1.2 Synthesis of Ru(Mebimpy)Cl_3

The complex was prepared following the method used for Ru(tpy)Cl_3, substituting Mebimpy for tpy.[2] In a typical synthesis, $RuCl_3 \cdot 3H_2O$ (1.00 g, 3.83 mmol) and Mebimpy (1.30 g, 3.83 mmol) were dissolved in 400 mL of ethanol and heated under reflux for 3 hours. After cooling to room temperature, the resulting brown precipitate was collected by filtration, washed thoroughly with ethanol until the washings were colorless, followed by ether.

1.3 Synthesis of [((Mebimpy)(Cl)Ru)$_2$$Cl_2$]

Ru(Mebimpy)Cl_3 (500 mg) was suspended in ethanol (40 mL), degassed with argon, then triethylamine (1.5 mL) was added. The mixture was refluxed for 2 hours, filtered hot, and the

purple solid washed with ethanol and ether to remove soluble $[Ru(Mebimpy)_2]Cl_2$ impurity. The product $[((Mebimpy)(Cl)Ru)_2Cl_2]$ was used without further purification.

1.4 Synthesis of $[Ru(Mebimpy)(dcbpy)H_2O]^{2+}$

The complex was synthesized following previously reported procedure.[3] Briefly, $[((Mebimpy)(Cl)Ru)_2Cl_2]$ (300 mg, 0.29 mmol), dcbpy (170 mg, 0.69 mmol), 0.1 g LiCl and 1 ml TEA were suspended in 100 mL of a 3:1 ethanol-water mixture and degassed with argon. The suspension was refluxed for 18 hours. The hot reaction mixture was filtered. The filtrate volume was reduced to 25 mL by rotary evaporation and 10 mL of a saturated aqueous NH_4PF_6 was added. 1 M HCl was added dropwise until $[Ru(Mebimpy)(dcbpy)H_2O]PF_6$ was precipitated. The resulting brown microcrystalline solid was collected by filtration and washed with water and ether and dried overnight under vacuum at 60 °C. ^1H NMR (400 MHz, CD_3OD) δ 11.32 (d, 1H), 9.04 – 8.91 (m, 2H), 8.76 (d, 2H), 8.55 (d, 1H), 8.28 (t, 1H), 8.05 (dd, 1H), 8.00 – 7.93 (m, 2H), 7.67 (d, 2H), 7.37 (t, 2H), 7.07 (t, 2H), 6.19 (d, 2H), 4.49 (s, 6H).

2. Fabrication of RuM-UiO-67 thin film

To prepare RuM-UiO-67 thin films, $ZrCl_4$ (58.25 mg, 0.25 mmol), $[Ru(Mebimpy)(dcbpy)H_2O]PF_6$ (34.2 mg, 0.043 mmol), and BPDC ligand (50.8 mg, 0.21 mmol) were dissolved in 10 mL of DMF in a 6-dram vial and sonicated until fully dissolved. One clean FTO slide was placed in the vial with the conductive side facing down. The FTO slides were cleaned by sequential sonication in alconox, deionized water, and acetone, then dried at 90 °C. The sealed vial was heated at 120 °C for 24 hours. After cooling, the films were washed with DMF and water, air-dried, and soaked in fresh water for 3 days to remove residual DMF. Finally, the films were dried in a vacuum oven and soaked in water for 24 hours.

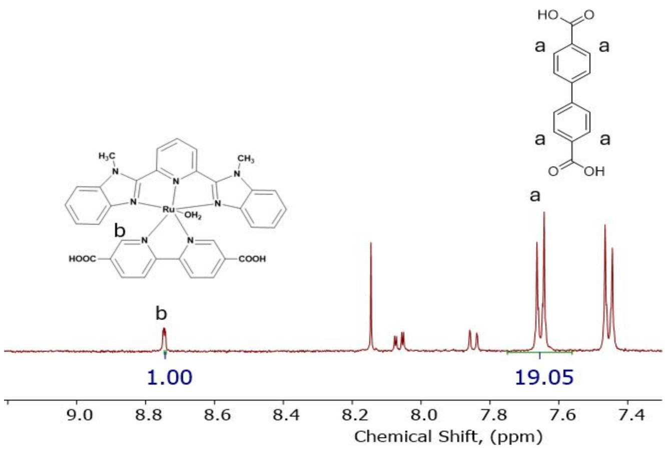

Figure S1. ^{1}H NMR spectrum of digested RuM-UiO-67.

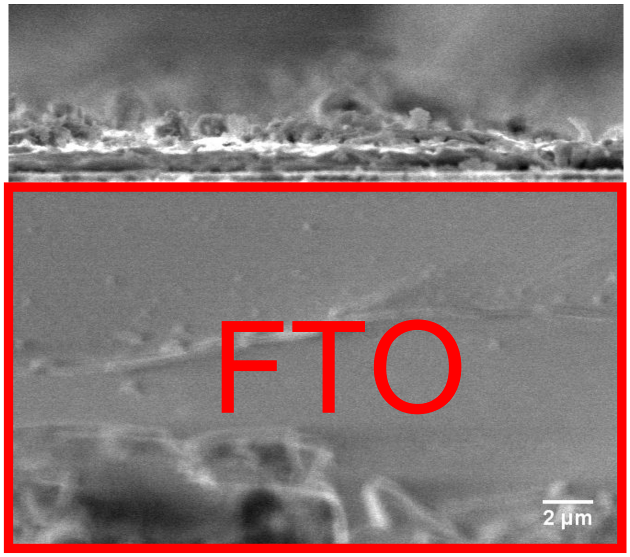

Figure S2. Cross-section SEM image of RuM-UiO-67 MOF thin film on FTO. The "FTO" designation denotes the fluorine-doped tin oxide substrate.

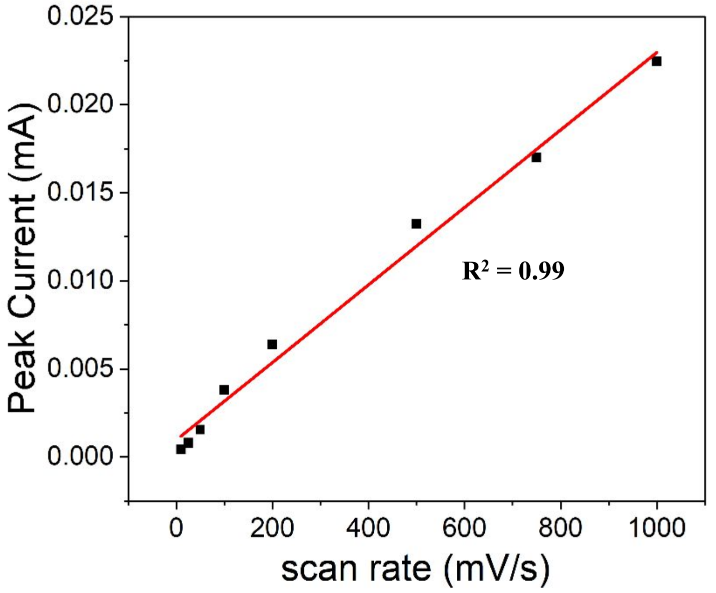

Figure S3. Peak current versus scan rate plot.

Electroactive Surface Area Comparison of RuM-UiO-67 : MOF vs Monolayer

Electroactive Surface Area (Γ) $\Gamma = \dfrac{Q}{nFA}$

Where Q = charge (in Coulombs), A = geometric area of the electrode (in cm^2), n =number of electrons

Q = 115.6 μC from cyclic voltammogram

n = 1e

F = 96485 C.mol^{-1}

A = 1 cm^2

Γ = 1.19×10^{-9} mol.cm^{-2}

Γ for monolayer of same catalyst = 1×10^{-10} mol.cm^{-2}

Electroactive site coverage of the film is more than ~12 times higher than that for a full packing monolayer catalyst on the same FTO electrode

Figure S4. Electroactive Surface Area Comparison of RuM-UiO-67: MOF vs Monolayer.

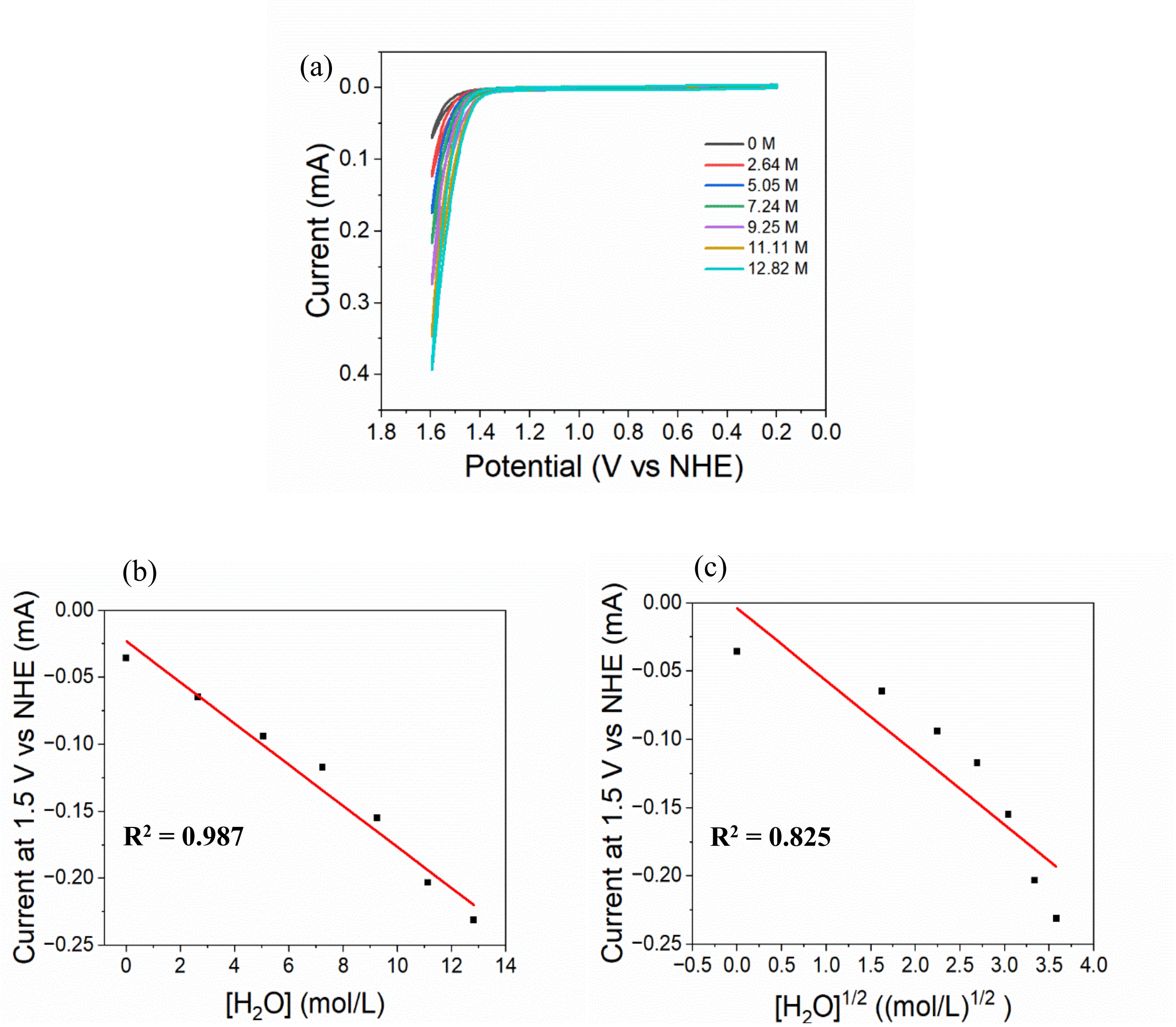

Figure S5. (a) The relationship between of catalytic current of RuM-UiO-67 vs $[H_2O]$ in 0.1 M $LiClO_4/CH_3CN$; (b) and (c), plot of the current at 1.5 V vs NHE as a function of $[H_2O]$ and $[H_2O]^{1/2}$, respectively.

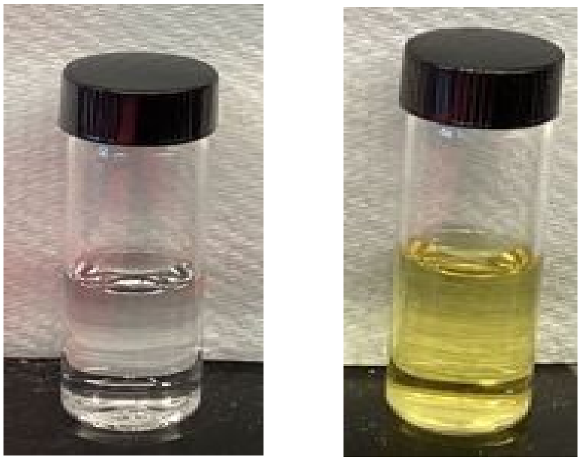

Figure S6. A KI–acetic acid aqueous solution was photographed before (left) and after (right) addition of an aliquot of the electrolyte collected following water-oxidation electrolysis.

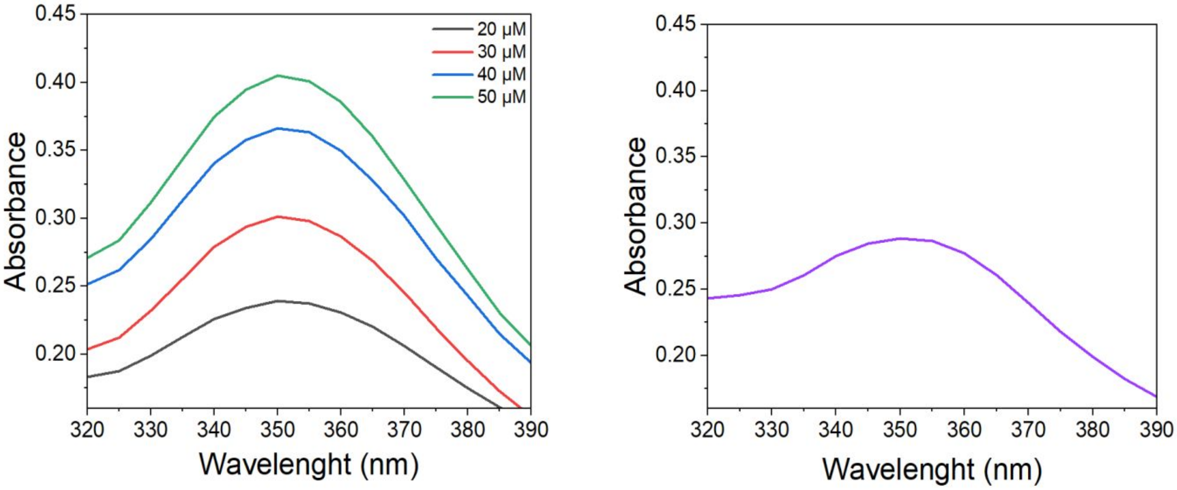

Figure S7. UV-vis spectra of increasing concentrations of standard H_2O_2 solutions (left) and post-catalysis solution (right) with the addition of fixed amount of potassium iodide.

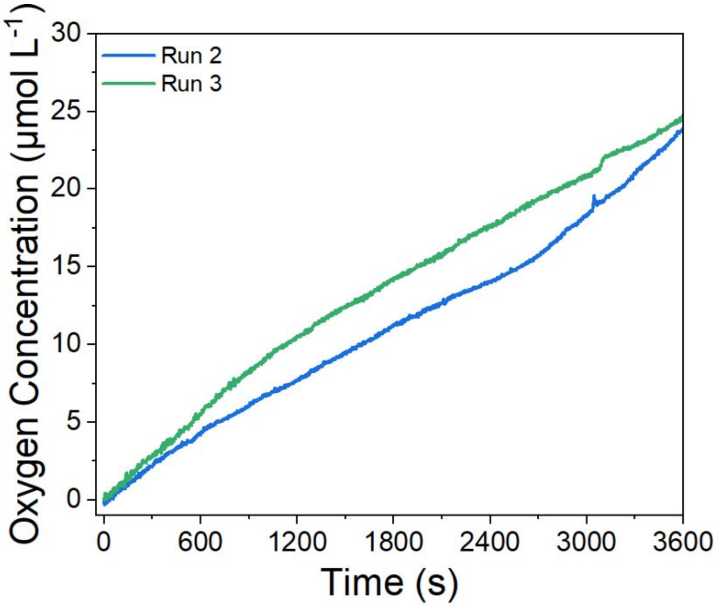

Figure S8. Repeated water-oxidation electrolysis of RuM-UiO-67 thin films.

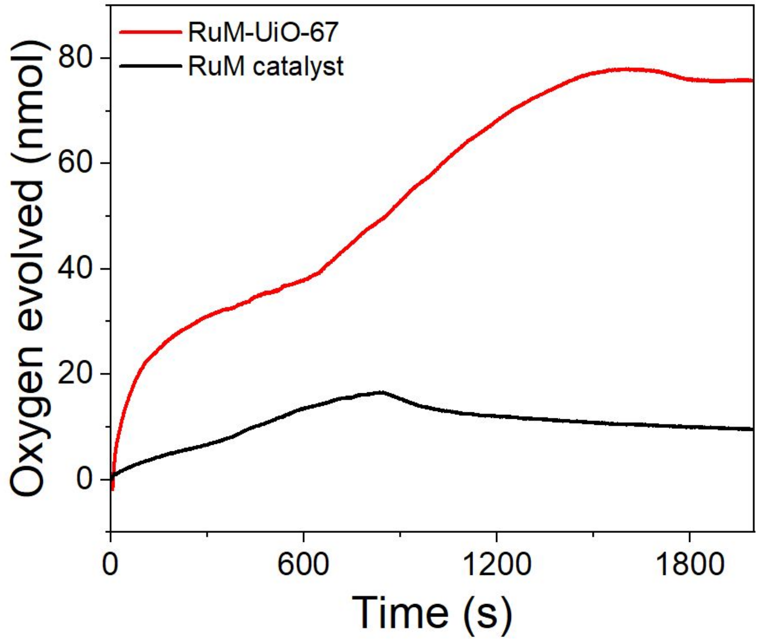

Figure S9. Oxygen evolution data for RuM-UiO-67 (red) and RuM catalyst (black).

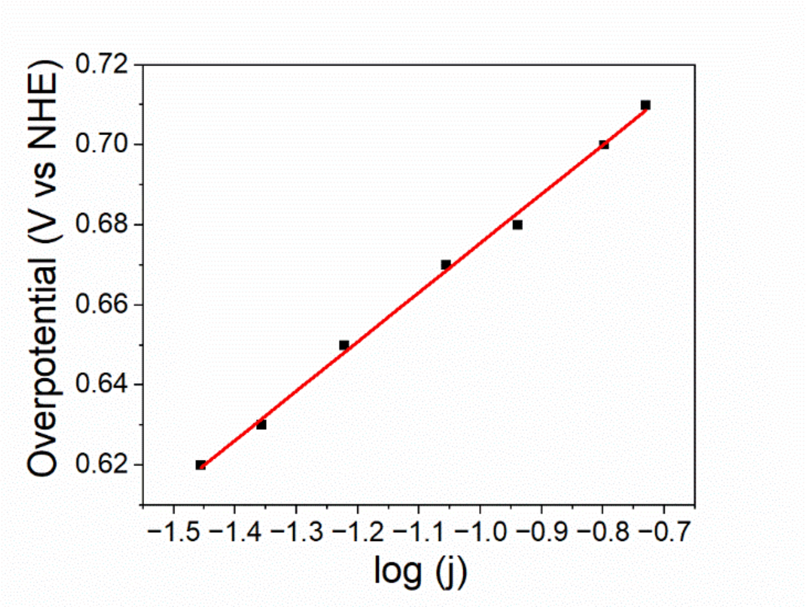

Figure S10. Tafel plot of RuM-UiO-67 in 0.1 M aqueous $LiClO_4$.

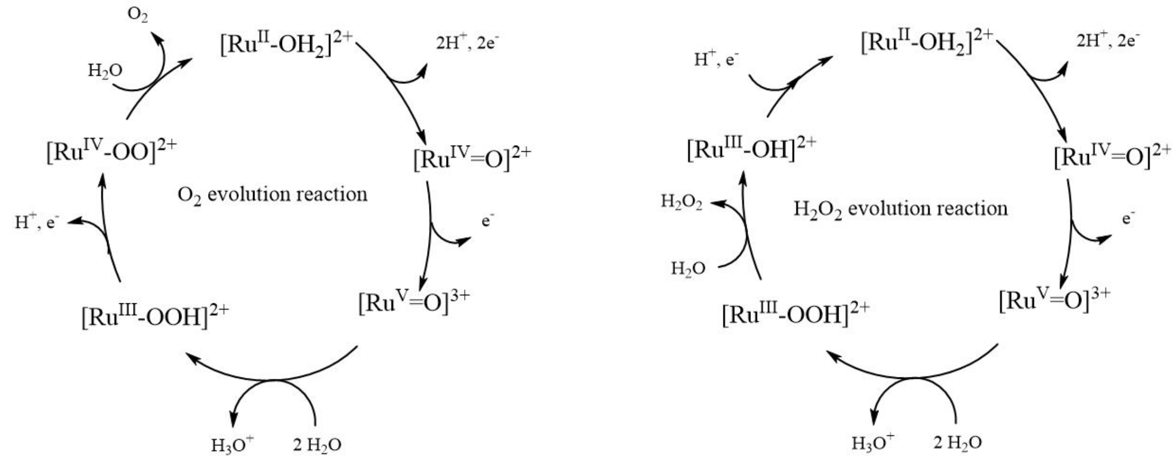

Figure S11. Probable mechanistic pathways for competitive water oxidation in RuM-UiO-67 MOF.

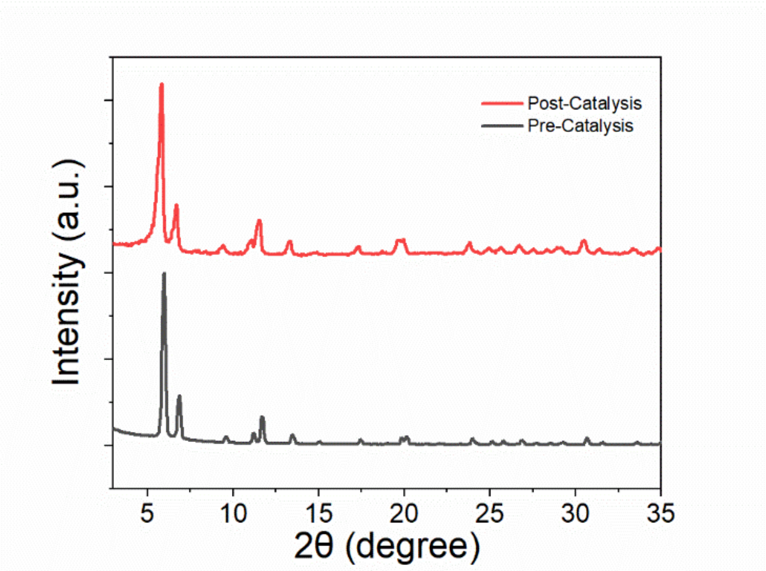

Figure S12. Powder X-ray Diffraction (PXRD) patterns of a RuM-UiO-67 film pre- and post-catalysis.

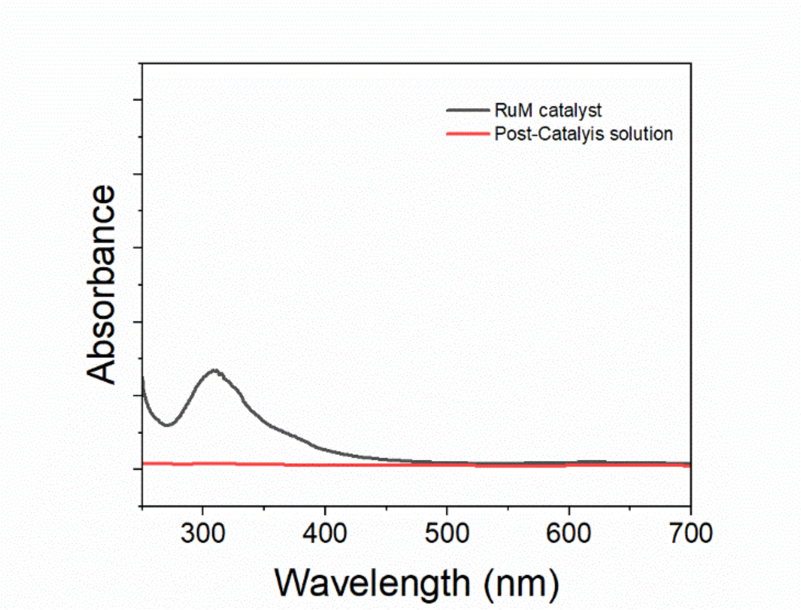

Figure S13. UV-Vis absorption spectra of 0.01 M molecular $[Ru(Mebimpy)(dcbpy)H_2O]^{2+}$ catalyst and the post-catalysis solution.

Table S1. Summary of literature-reported diffusion coefficients for MOFs and comparison with the RuM–UiO-67 system studied here.

Redox-active MOFs	Apparent Diffusion Coefficient (cm^2/s)	References
UiO67-[$RuOH_2$]@FTO [Ru(tpy)(dcbpy)OH_2](ClO_4)$_2$ (tpy = 2,2':6',2"-terpyridine, dcbpy = 2,2'-bipyridine-5,5'-dicarboxylic acid)]	9.56×10^{-11}	4
Ir-UiO-66	10^{-12}	5
RuBPY-UiO-67 [RuBPY = Ru(bpy)$_2$(bpy-(COOH)$_2$) (bpy = 2,2′-bipyridine; bpy-(COOH)$_2$ = 5,5′-dicarboxylic acid-2,2′-bipyridine)]	$8(\pm3)\times10^{-9}$	6
NU-1000	2×10^{-10}	7
Zr-(dcphOH-NDI) [NDI = naphthalene diimide]	10^{-11}	8
RuM-UiO-67	$(5 \pm 1.3)\times10^{-11}$	This Work

References

(1) Xu, X.; Xi, Z.; Chen, W.; Wang, D. Synthesis and Structural Characterization of Copper(II) Complexes of Pincer Ligands Derived from Benzimidazole. *J. Coord. Chem.* **2007**, *60* (21), 2297–2308. https://doi.org/10.1080/00958970701261352.

(2) Gibbons, B.; Cairnie, D. R.; Thomas, B.; Yang, X.; Ilic, S.; Morris, A. J. Photoelectrochemical Water Oxidation by a MOF/Semiconductor Composite. *Chem. Sci.* **2023**, *14* (18), 4672–4680. https://doi.org/10.1039/D2SC06361A.

(3) Concepcion, J. J.; Jurss, J. W.; Norris, M. R.; Chen, Z.; Templeton, J. L.; Meyer, T. J. Catalytic Water Oxidation by Single-Site Ruthenium Catalysts. *Inorg. Chem.* **2010**, *49* (4), 1277–1279. https://doi.org/10.1021/ic901437e.

(4) Johnson, B. A.; Bhunia, A.; Ott, S. Electrocatalytic Water Oxidation by a Molecular Catalyst Incorporated into a Metal–Organic Framework Thin Film. *Dalton Trans.* **2017**, *46* (5), 1382–1388. https://doi.org/10.1039/C6DT03718F.

(5) Chuang, C.-H.; Li, J.-H.; Chen, Y.-C.; Wang, Y.-S.; Kung, C.-W. Redox-Hopping and Electrochemical Behaviors of Metal–Organic Framework Thin Films Fabricated by Various Approaches. *J. Phys. Chem. C* **2020**, *124* (38), 20854–20863. https://doi.org/10.1021/acs.jpcc.0c03873.
(6) Thomas, B.; Basak, S.; Smith, Q.; Yan, M.; Morris, A. J. Rapid Redox Hopping Charge Transfer and Electrochromism in a Multivariate Metal–Organic Framework. *J. Am. Chem. Soc.* **2025**, *147* (37), 33655–33665. https://doi.org/10.1021/jacs.5c09275.
(7) Goswami, S.; Hod, I.; Duan, J. D.; Kung, C.-W.; Rimoldi, M.; Malliakas, C. D.; Palmer, R. H.; Farha, O. K.; Hupp, J. T. Anisotropic Redox Conductivity within a Metal–Organic Framework Material. *J. Am. Chem. Soc.* **2019**, *141* (44), 17696–17702. https://doi.org/10.1021/jacs.9b07658.
(8) Johnson, B. A.; Bhunia, A.; Fei, H.; Cohen, S. M.; Ott, S. Development of a UiO-Type Thin Film Electrocatalysis Platform with Redox-Active Linkers. *J. Am. Chem. Soc.* **2018**, *140* (8), 2985–2994. https://doi.org/10.1021/jacs.7b13077.
